# Adherence to Mediterranean Diet and NAFLD in Patients with Metabolic Syndrome: The FLIPAN Study

**DOI:** 10.3390/nu14153186

**Published:** 2022-08-03

**Authors:** Sofía Montemayor, Catalina M. Mascaró, Lucía Ugarriza, Miguel Casares, Isabel Llompart, Itziar Abete, María Ángeles Zulet, J. Alfredo Martínez, Josep A. Tur, Cristina Bouzas

**Affiliations:** 1 Research Group on Community Nutrition and Oxidative Stress, University of the Balearic Islands-IUNICS, 07122 Palma de Mallorca, Spain; sofiamf16@gmail.com (S.M.); c.mascaro@uib.es (C.M.M.); luciaugarriza@gmail.com (L.U.); isabel.llompart@ssib.es (I.L.); cristina.bouzas@uib.es (C.B.); 2 Health Institute of the Balearic Islands (IDISBA), 07120 Palma de Mallorca, Spain; 3 Camp Redó Primary Health Care Center, 07010 Palma de Mallorca, Spain; 4 Radiodiagnosis Service, Red Asistencial Juaneda, 07011 Palma de Mallorca, Spain; casaresmiguel@gmail.com; 5 Clinical Analysis Service, University Hospital Son Espases, 07120 Palma de Mallorca, Spain; 6 CIBEROBN (Physiopathology of Obesity and Nutrition CB12/03/30038), Instituto de Salud Carlos III (ISCIII), 28029 Madrid, Spain; iabetego@unav.es (I.A.); mazulet@unav.es (M.Á.Z.); 7 Department of Nutrition, Food Sciences, and Physiology, Center for Nutrition Research, University of Navarra, 31008 Pamplona, Spain; jalfredo.martinez@imdea.org; 8 Cardiometabolics Precision Nutrition Program, IMDEA Food, CEI UAM-CSIC, 28049 Madrid, Spain

**Keywords:** Mediterranean diet, NAFLD, non-alcoholic fat liver disease, intrahepatic fat contents, metabolic syndrome

## Abstract

Unhealthy diet is an important factor in the progression of non-alcoholic fatty liver disease (NAFLD). Previous studies showed the benefits of a Mediterranean diet (MedDiet) on Metabolic syndrome (MetS), type 2 diabetes mellitus (T2DM), and cardiovascular diseases, which usually have a pathophysiological relationship with NAFLD. To assess the effect of adherence to a MedDiet on NAFLD in MetS patients after lifestyle intervention, this multicentre (Mallorca and Navarra, Spain) prospective randomized trial, with personalized nutritional intervention based on a customized MedDiet, coupled with physical activity promotion was performed to prevent, and reverse NAFLD among patients with MetS. The current analysis included 138 patients aged 40 to 60 years old, Body Mass Index (BMI) 27–40 kg/m^2^, diagnosed with NAFLD using MRI, and MetS according to the International Diabetes Federation (IDF). A validated food frequency questionnaire was used to assess dietary intake. Adherence to Mediterranean diet by means of a 17-item validated questionnaire, anthropometrics, physical activity, blood pressure, blood biochemical parameters, and intrahepatic fat contents (IFC) were measured. The independent variable used was changes in MedDiet adherence, categorized in tertiles after 6 months follow-up. Subjects with high adherence to the MedDiet showed higher decreases in BMI, body weight, WC, SBP, DBP, and IFC. An association between improvement in adherence to the MedDiet and amelioration of IFC after 6-month follow-up was observed. High adherence to the MedDiet is associated with better status of MetS features, and better values of IFC.

## 1. Introduction

Non-alcoholic fatty liver disease (NAFLD) is defined as the accumulation of excessive fat in the liver not linked to alcohol abuse [1], and is associated with high levels of liver enzymes, insulin resistance, type 2 diabetes mellitus (T2DM) and cardiovascular risk [2]. It encompasses a spectrum of histopathological features, ranging from simple non-alcoholic fatty liver (NAFL) to non-alcoholic steatohepatitis (NASH), progressing to liver fibrosis, cirrhosis, and ultimately to hepatocellular carcinoma [3]. Metabolic syndrome (MetS) is characterized by abdominal obesity, insulin resistance, hypertension, and hyperlipidaemia [4]. MetS was diagnosed after a minimum of three out of five criteria were confirmed according to the International Diabetes Federation, the American Heart Association and the National Heart, Lung, and Blood Institute [5]. NAFLD diagnostic profile is like that of MetS, such as abdominal obesity, high triacylglyceridemia (TG), low high-density lipoprotein (HDL) cholesterol, high blood pressure, and increased fasting glycemia [6]. These biomarkers are usually linked to unhealthy diet and lifestyle, which can lead to liver damage [7]. 

Unhealthy dietary composition is an important factor in the progression of non-alcoholic fatty liver disease (NAFLD). Weight loss by lifestyle changes is the current evidence-based therapy strategy for nutrition [8]. Guidelines like the American Association for the Study of Liver Disease (AASLD) have not made any specific dietary recommendation for NAFLD. The European Association for the Study of the Liver (EASL) recommend the exclusion of components promoting NAFLD, in addition to a macronutrient composition in line with a Mediterranean diet. Nevertheless, this recommendation is only supported by evidence graded as “moderate” in quality [9]. In a recent review it was concluded that data is supportive for diets reduced in saturated fatty acids, processed meats, and refined carbohydrates. Plant-based diets like the DASH, Mediterranean, vegetarian, and vegan diets incorporate these suggestions [10]. There is research that supports particular diets, most notably the Mediterranean diet (MedDiet) [11]. The MedDiet is defined as a plant-based diet characterized by a high intake of fruits and vegetables, legumes, whole grains, and a high ratio of monounsaturated fatty acids (MUFA), which is associated with a lower risk of many chronic diseases [12]. Studies have shown the benefits of the MedDiet in conditions including MetS, T2DM, and cardiovascular diseases. These conditions usually co-exist, have a pathophysiological relationship with NAFLD [13], and it was even suggested a reduced NAFLD severity followed from an adherence to this dietary pattern [14]. In addition, adherence to the MedDiet has also been linked to less advanced NAFLD and a lower risk of having the metabolic syndrome in this patient population [15]. 

There is strong evidence that points toward recommending the MedDiet for NAFLD [16]. Nevertheless, the existing evidence regarding the optimal diet composition for NAFLD remains conflicting [17]. However, the adherence to the MedDiet seems to play an important role in improving liver steatosis and metabolic dysfunctions in this patient population. Therefore, the aim of this paper was to assess the effect of adherence to the MedDiet on NAFLD on MetS patients after lifestyle intervention.

## 2. Methods

### 2.1. Design

This is a multicentre (Mallorca and Navarra, Spain) prospective randomized trial, with personalized nutritional intervention based on a customized MedDiet, coupled with physical activity promotion to prevent, and reverse NAFLD among patients with MetS. Protocol details are in ClinicalTrials.gov (https://clinicaltrials.gov/ct2/show/NCT04442620 (accessed on 21 April 2022)) with number NCT04442620 [18]. The study protocol was reviewed and approved by the Ethics Committee of the Balearic Islands (ref. IB 2251/14 PI) and the Ethics Committee of the University of Navarra (ref. 054/2015mod2), and it followed the Declaration of Helsinki ethical standards. All participants were informed of the purpose and the implications of the study and provided the written consent to participate.

### 2.2. Subjects

The current analysis included patients aged 40 to 60 years old. They all had a Body Mass Index (BMI) of 27–40 kg/m^2^, were diagnosed with NAFLD using magnetic resonance imaging (MRI), and achieved MetS criteria according to the International Diabetes Federation (IDF) [19]. Exclusion criteria were previous cardiovascular disease, liver disease (other than NAFLD), cancer or a history of malignancy in the previous 5 years, haemochromatosis, previous bariatric surgery, non-medicated depression, alcohol and drug abuse, pregnancy, primary endocrinological diseases (other than non-medicated hypothyroidism), severe psychiatric disorders (schizophrenia, bipolar disorder, eating disorders, or depression with hospitalization within the last 6 months) or a Beck Depression Inventory score >30, concomitant therapy with steroids, or inability to provide informed consent. The adults who met the selection criteria (n = 155) were randomized as previously described [20]. Five participants withdrew the consent before intervention, and 22 did not complete the validated 17-item MedDiet adherence questionnaire needed for the present manuscript [21]. The final sample was constituted by 128 subjects.

### 2.3. Dietary Intakes

The subjects followed the customized dietary and physical activity interventions previously described [20] for 6 months, and each intervention aimed at reducing caloric intake by 25–30% of the baseline calories intake and increase energy expenditure by 400 kcal/70 kg per day (5.7 kcal per kg of body weight) for the physical activity component. A daily calorie prescription was provided to patients by well-trained dieticians, as well as dietary plans based on exchange systems, and a 7-day menu with menus adapted to seasonal food. Dietary intake was collected at baseline and 6-months by means of a validated 148 items-Food Frequency Questionnaire (FFQ) [22]. The items represent the consumption of usual food and drink portion sizes, and participants were asked how often they consumed the amount of an item reported on the FFQ during the past 12 months. Answers could be never or less than once per month to six or more times per day. Foods not included in the questionnaire were manually entered. Energy and nutrients intakes were calculated from each food or drink using a computer program based on food composition tables [23]. Dietary information from the FFQ included total energy expressed as kcal per day, macro- and micro-nutrient daily intake, and provided information according to food groups.

### 2.4. Adherence to Mediterranean Diet

Adherence to the MedDiet was assessed by means of a validated 17-item MedDiet adherence questionnaire previously used [21], which all participants answered. A score was given for each met objective: 1 (compliance) or 0 (non-compliance). The total score ranged between 0 and 17, such as a score of 0 indicated no compliance, and a score of 17 indicated maximum adherence. 

The independent variable used was changes in adherence to the MedDiet after a 6-month follow-up. This variable was obtained by subtracting the score at 6 months to the score at baseline. The obtained changes in adherence to the MedDiet were categorized in tertiles. Therefore, participants were classified in three groups: no changes of adherence to MedDiet (1st tertile, ≤0 points, including negative values); moderate changes in adherence to MedDiet (2nd tertile, ≥1 to 4 points); high changes of adherence to MedDiet (3rd tertile, ≥5 points).

### 2.5. Anthropometrics and Blood Pressure

Weight, body fat, body mass index (kg/m^2^, BMI), waist circumference (WC), blood pressure (BP), and energy expenditure were obtained. A mobile stadiometer (Seca 213, SECA Deutschland, Hamburg, Germany) was used to measure height to the nearest millimetre at baseline, with the participant’s head maintained in the Frankfort plane. A Segmental Body Composition Analyzer for impedance testing (Tanita MC780P-MA, Tanita, Tokyo, Japan) was used to measure body weight, with participants wearing light clothes and without shoes (0.6 kg of weight was subtracted for their clothing). The waist circumference was taken halfway between the last rib and the iliac crest, with participants standing upright, using a measuring tape. BMI was calculated as weight in kg/height in m^2^, using the standard formula. A validated semi-automatic oscillometer (Omron HEM-705CP, Hoofddorp, The Netherlands) was used to measure BP in triplicate (2 min apart) in the non-dominant arm after a 5-min rest in a seated position. The average of the three measurements was used for statistics. 

### 2.6. Blood Collection and Analysis

At baseline and 6 months, venous blood and single spot urine samples were collected in the morning after a 12-h overnight fast. Blood was extracted into vacutainers containing ethylenediamine tetra-acetic acid (EDTA), citrate, or serum before immediate centrifugation at 3000 rpm for 10 min. Measures, which included fasting glucose, aspartate aminotransferase (AST), alanine aminotransferase (ALT), gamma-glutamyl transferase (GGT), high-density lipoprotein cholesterol (HDL-C), and triglyceride (TG) were measured in serum on the Abbott ARCHITECT c16000 employing commercial kits (Abbott Diagnostics, Lake Forest, IL, USA). The insulin resistance index was calculated using the Homeostatic Model Assessment for Insulin Resistance (HOMA-IR) formula [24].

### 2.7. Intrahepatic Fat Contents Assessment

Abdominal MRI (Signa Explorer 1.5T, General Electric Healthcare, Chicago, IL, USA) was used to quantify liver fat as mean percentage (%). A mean intrahepatic fat contents (IFC) ≥6.4% was considered clinically relevant [25]. In the current paper, authors considered that a reversion of IFC occurred when subjects lowered their mean IFC to ≥6.4%, and an amelioration of the liver was when subjects could lower their mean IFC from 1 point and on of change of the percentage.

### 2.8. Statistics

The sample size was estimated for weight loss as the primary outcome of the study, assuming a two-group *t*-test (two-sided) of the difference between the control group and the two intervention groups (group ratio = 2). Based on previous evidence [26,27], a weight reduction difference of 2.5 kg with a standard deviation (SD) of 4.5 was expected between the control group and the intervention groups. A total sample size of 150 patients was needed to give the trial a 95% power to detect a statistically significant difference in weight loss between the control and the intervention groups (α = 0.05), as well as to account for a 20% drop-out rate. The analysis was conducted by modified intention to treat (mITT) with randomized participants analyzed according to the treatment group. The SPSS statistical software package, version 25.0 (SPSS Inc., Chicago, IL, USA) was used. Chi-square was used for the sociodemographic characteristics of the subjects at baseline according to tertiles of changes in adherence of the MedDiet. Continuous variables were expressed as means ± standard deviation (SD). Categorical data were expressed as count and percentage. Normality distributions for continuous data were determined using visual inspection of histograms and normal probability plots. One-way analysis of variance (ANOVA) was used to test clinical and diet features after a 6-month follow-up by tertiles of changes in adherence to the MedDiet. One-way ANCOVA was used was to assess the changes between groups, controlling for baseline measures. The Bonferroni method was used to perform post-hoc analyses. Finally, a multivariable logistic regression analysis was used to investigate if the tertiles of changes in adherence could lead to a reversion to normal values of IFC, and an amelioration of the liver after 6 months follow-up. The following covariates were entered in the model: waist circumference (continuous), hypertriglyceridemia (triglyceridemia ≥150 mg/dL), high blood pressure (130/85 mmHg or higher), T2DM (yes/no). All *p*-values were two-sided, with *p* < 0.05.

## 3. Results

Table 1 shows the sociodemographic characteristics of participants according to tertiles of changes of adherence to the MedDiet at baseline. No differences between groups were registered in the considered sociodemographic and clinical parameters.

Table 2 shows the clinical features of participants according to the changes of adherence to MedDiet at 6-month follow-up. It was observed an improvement of subjects’ clinical parameters on the High changes in the adherence to the MedDiet group. High changes in adherence to the MedDiet group showed a higher decrease in BMI, body weight, WC, SBP, DBP, and IFC than in Moderate changes group and No changes group. TG was slightly higher in High changes group than in Moderate changes group.

Table 3 shows the dietary features of participants, according to the changes of adherence to the MedDiet at a 6-month follow-up. No changes in adherence to MedDiet group decreased in the adherence, in contrast with the increase observed in the Moderate changes group and High changes group. High increases in nuts and dairy consumption, and high decreases in soft drinks consumption were observed mainly in High changes group, intermediate increases and decreases in Moderate changes group, and minimal changes in No changes MedDiet group. 

Table 4 shows the association between the reversion and amelioration of intrahepatic fat contents (IFC) and the changes of adherence to the MedDiet after a 6-month follow-up. It was observed that there is a direct association (crude OR) between changes to adherence to the MedDiet, and reversion and amelioration of IFC. After adjustment by waist circumference, high blood pressure, and type 2 diabetes mellitus, just amelioration of IFC was significant.

## 4. Discussion

The results of the present analysis showed an association between an improvement in the adherence to the MedDiet after a 6-month follow-up and better status of MetS features, as well as better values of IFC by MRI. Subjects with high changes in adherence to the MedDiet showed lower IFC at the 6-month follow-up, and this high adherence to the MedDiet was associated with amelioration of IFC.

These findings are consistent with literature pointing out that the MedDiet has preventive effect on NAFLD [28]. A study conducted in Australia found that following the MedDiet for 6 weeks resulted in reversal of NAFLD by lowering intrahepatic lipids (−39%) and decreasing insulin resistance (−1.7 mmol/L, using HOMA-IR); they found that MedDiet decreases the intrahepatic fat contents independently from weight loss [29]. All these findings showed that the MedDiet could be recommended in NAFLD patients.

The current study reported that patients with high changes in adherence to MedDiet had lower body weight, WC, BMI, TG, SBP, DBP and IFC, which are in line with a previous study suggesting that the MedDiet had benefits on MetS, T2DM and cardiovascular diseases, which are pathophysiologically linked with NAFLD [13]. According to previous studies, an increased adherence to a MedDiet in patients with NAFLD can improve intrahepatic lipid levels, fibrosis, insulin resistance, inflammatory markers, and other metabolic risk markers [30,31,32,33]. 

The current study also showed how specific consumption of food groups has increased or decreased. The soft drinks consumption was lowered as the adherence to the MedDiet increased. Nuts consumption was improved as the adherence to the MedDiet increased, and interestingly, dairy consumption was increased too in the High changes group. The food groups highlighted on high adherence could reverse the intrahepatic fat contents.

Current findings are in line with the literature. In patients with NAFLD, daily fructose ingestion, due to industrial rather than fruit-derived production, is associated with increased fibrosis [34,35]. Sweetened beverages that contain fructose-containing sugars such as sucrose and high fructose corn syrup are associated with increased risk of developing steatosis and NASH, especially in overweight/obese individuals [36]. As for nuts, these are nutritionally dense fruits, consisting of a unique blend of fatty acids, bioactive compounds, and essential nutrients [37]. A regular weekly consumption is associated with decreased incidence of obesity, T2DM, hypertension and the metabolic syndrome [38]. In addition, nuts show much therapeutic potential in treating patients with NAFLD through improvements to lipid profile, hepatic steatosis, and inflammation [39]. Regarding dairy, the reviewed MedDiet pyramid depiction emphasizes on consuming 2 servings of dairy and preferably low fat [12]. Dairy products, including milk, yogurt, and cheese are nutrient-dense foods that influence health outcomes via gut microbiota composition [40]. A meta-analysis showed the therapeutic value of dairy proteins, especially low-fat dairy products, with beneficial effects on metabolic syndrome, insulin resistance, waist circumference, and body weight [41,42]. Moreover, a study made in middle-aged and older Korean adults, found that higher dairy protein intake is associated with lower incidences of NAFLD [43].

It has been currently found that high adherence to the MedDiet could reverse the IFC. A meta-analysis conducted with Italian and Spanish data suggested that a possible explanation for the success of the MedDiet was successful on human health, is because of the synergic effect of all the MedDiet components, rather than the individual food or nutrient intake [44]. The MedDiet was associated with an overall better metabolic status due to a higher profile of the MUFA:SFA ratio, as well as to the high contents in fiber, antioxidants, and polyphenols with anti-inflammatory properties [44]. 

Increased WC shows visceral fat accumulation, which causes hepatic inflammation and oxidative damage, both of which promote hepatic steatosis [45]. A previous study on 1500 subjects found that keeping the waist circumference in a normal range can prevent fatty liver, even if a proper diet is not followed [46]. So, high compliance to the MedDiet could help to maintain WC at normal levels, helping visceral fat to not accumulate in the liver [47].

### Strengths and Limitations

The current study increases the limited evidence for the MedDiet and the reversion of the fatty liver disease in subjects with MetS and was studied in a greater number of patients than previous studies [45]. Despite it, the study has been conducted in a limited number of patients, which could be considered as a limitation of the study. Another limitation was the estimated values of the FFQ even after being validated, which might overestimate intake of certain food groups [48].

## 5. Conclusions

High adherence to the MedDiet is associated with an amelioration of intrahepatic fat contents, together with better status of MetS features. Thus, a greater adherence to a Mediterranean dietary pattern may be beneficial, besides helping the metabolic syndrome status, diabetes, and other diseases, to reverse a condition of the fatty liver disease. Nevertheless, prospective longitudinal cohort studies are required with larger sample sizes, to determine dietary patterns, intakes, and specific food groups to reverse the conditions of NAFLD, and MetS.

## Figures and Tables

**Table 1:** Baseline sociodemographic characteristics according to tertiles of MedDiet adherence changes after 6-month follow-up.

Outcomes	No Changes in Adherence to MedDiet (n = 15)	Moderate Changes in Adherence to MedDiet (n = 56)	High Changes in Adherence to MedDiet (n = 57)	*p* *
Age (y) (mean ± SD)	53.3 ± 7.2	53.5 ± 8.0	52.0 ± 6.9	0.516
Marital Status (n; %)				0.402
Single	0 (0.0)	4 (3.1)	8 (6.3)	
Married/domestic partnership	12 (9.4)	41 (32.0)	44 (33.6)	
Divorced/separated/widowed	3 (2.3)	11 (8.6)	6 (4.7)	
Employment (n; %)				0.338
Working	11 (8.6)	38 (29.7)	44 (34.4)	
Unemployed/retired/housewife	4 (3.1)	18 (14.1)	13 (10.2)	
Education Level (n; %)				0.219
University/post-university	4 (3.1)	4 (3.1)	2 (1.6)	
Secondary education	7 (5.5)	24 (18.8)	24 (18.8)	
Primary education	2 (1.6)	24 (18.8)	29 (22.7)	
None	2 (1.6)	4 (3.1)	2 (1.6)	
Currently smoking (n; %)	0 (0.0)	7 (5.3)	9 (6.8)	0.554
Regular Physical Activity (n; %)				0.246
None	4 (2.9)	23 (16.7)	32 (23.2)	
Light	6 (4.3)	23 (16.7)	19 (13.8)	
Moderate	5 (3.6)	7 (5.1)	10 (7.2)	
Heavy	0 (0.0)	3 (2.2)	6 (4.3)	
T2DM (%)	4 (2.9)	23 (33.8)	11 (16.2)	0.141
High BP (n; %)	4 (2.9)	17 (12.2)	9 (6.5)	0.062

Abbreviations: BP: blood pressure; SD: standard deviation; T2DM: type 2 diabetes mellitus. * By chi-square.

**Table 2:** Six-month follow-up changes of clinical features according to the changes in adherence to MedDiet.

Outcomes		No Changes in Adherence to MedDiet (n = 15)	Moderate Changes in Adherence to MedDiet (n = 56)	High Changes in Adherence to MedDiet (n = 57)	*p* ^‡^
BMI (kg/m^2^)	Basal	32.6 ± 3.0	33.6 ± 3.5	33.9 ± 3.9	<0.001
	6-months	31.9 ± 2.7	32.1 ± 3.6	30.9 ± 3.9	
	Δ	−0.7 ± 1.2 *^c^	−1.5 ± 1.6 *^b^	−3.0 ± 2.0 *^bc^	
Body weight (kg)	Basal	91.5 ± 14.8	94.7 ± 12.4	96.5 ± 14.5	<0.001
	6-months	89.5 ± 14.5	90.5 ± 12.4	88.1 ± 14.5	
	Δ	−1.9 ± 3.6 ^c^	−4.2 ± 4.6 *^b^	−8.5 ± 5.8 *^bc^	
WC (cm)	Basal	110.2 ± 9.4	113.0 ± 9.0	111.4 ± 9.0	0.004
	6-months	108.6 ± 7.4	110.5 ± 17.0	103.1 ± 10.5	
	Δ	−1.6 ± 4.1	−2.5 ± 15.1 ^b^	−8.3 ± 5.6 *^b^	
SBP (mmHg)	Basal	134.2 ± 19.4	138.0 ± 16.2	134.7 ± 13.9	0.013
	6-months	134.4 ± 16.5	134.1 ± 15.3	126.3 ± 15.0	
	Δ	+0.2 ± 11.7	−4.0 ± 16.7 ^b^	−8.4 ± 14.6 *^b^	
DBP (mmHg)	Basal	82.0 ± 8.6	85.2 ± 9.4	86.8 ± 8.8	0.009
	6-months	82.9 ± 6.8	82.7 ± 8.8	80.3 ± 9.0	
	Δ	+0.9 ± 7.4 ^c^	−2.6 ± 7.4 *^b^	−6.5 ± 8.9 *^bc^	
Glucose (mg/dL)	Basal	107.1 ± 16.6	120.1 ± 48.8	109.1 ± 20.9	0.079
	6-months	103.9 ± 14.3	116.8 ± 54.3	98.9 ± 16.9	
	Δ	−3.2 ± 15.3	−3.4 ± 25.4	−10.2 ± 15.5 *	
TG (mg/dL)	Basal	151.7 ± 74.0	224.0 ± 300.1	177.8 ± 76.5	0.020
	6-months	170.9 ± 135.0	175.1 ± 107.1	125.0 ± 76.3	
	Δ	+19.1 ± 132.5	−48.9 ± 70.3 *^b^	52.9 ± 70.4 *^b^	
HDL-chol (mg/dL)	Basal	47.8 ± 12.4	42.6 ± 10.0	45.5 ± 11.9	0.185
	6-months	47.6 ± 14.5	44.7 ± 10.7	48.8 ± 12.2	
	Δ	+0.2 ± 4.3	+2.1 ± 5.7 *	+3.3 ± 7.8 *	
HOMA-IR	Basal	5.8 ± 3.8	6.6 ± 4.7	5.2 ± 2.9	0.117
	6-months	5.1 ± 3.6	5.8 ± 7.3	3.1 ± 2.2	
	Δ	−0.7 ± 2.5	−0.8 ± 5.9	−2.1 ± 2.6 *	
Intrahepatic fat contents (%)	Basal	15.1 ± 13.9	13.4 ± 8.8	12.5 ± 12.2	0.003
	6-months	10.6 ± 7.3	9.7 ± 6.7	6.0 ± 6.4	
	Δ	−4.5 ± 14.2	−3.7 ± 5.8 *^b^	−6.5 ± 10.0 *^b^	
AST (U/L)	Basal	25.2 ± 12.0	24.0 ± 8.4	27.6 ± 15.7	0.639
	6-months	20.9 ± 5.1	22.9 ± 6.5	23.8 ± 13.2	
	Δ	−4.3 ± 10.1	−1.1 ± 7.2	−3.8 ± 14.2 *	
ALT (U/L)	Basal	33.8 ± 25.4	32.4 ± 17.8	40.9 ± 38.0	0.260
	6-months	24.5 ± 11.3	27.0 ± 14.3	26.0 ± 19.9	
	Δ	−9.3 ± 17.0	−5.3 ± 14.0 *	−14.9 ± 30.1 *	
GGT (U/L)	Basal	36.9 ± 27.1	44.1 ± 22.6	53.8 ± 71.1	0.197
	6-months	29.5 ± 15.5	40.0 ± 36.0	35.0 ± 56.1	
	Δ	−7.4 ± 14.1	−4.1 ± 24.3	−18.8 ± 50.0 *	

Abbreviations: ∆: delta; ALT: alanine aminotransferase; AST: aspartate aminotransferase; BMI: body mass index; DBP: diastolic blood pressure; GGT: gamma-glutamyl transferase; HDL-chol: high-density lipoprotein cholesterol; HOMA-IR: Homeostatic Model Assessment for Insulin Resistance; SBP: Systolic blood pressure; TG: triglycerides; WC: Waist circumference. Data are expressed as mean ± standard deviation (SD). * *p* < 0.05. ^‡^ Differences between tertile groups at 6 months after adjustment for baseline values by ANCOVA. Post hoc test by Bonferroni: ^a^: differences between No changes in MedDiet adherence and Moderate changes in MedDiet adherence; ^b^: differences between Moderate changes in MedDiet adherence and High changes in MedDiet adherence; ^c^: differences between No changes in MedDiet adherence and High changes in MedDiet adherence.

**Table 3:** Six-month follow-up changes on dietary features according to changes in adherence to MedDiet.

Outcomes		No Changes in Adherence to MedDiet (n = 15)	Moderate Changes in Adherence to MedDiet (n = 56)	High Changes in Adherence to MedDiet (n = 57)	*p* ^‡^
Adherence to MedDiet	Basal	9.0 ± 2.4	8.2 ± 2.6	6.2 ± 2.0	<0.001
	6-months	7.9 ± 2.2	10.9 ± 2.7	13.7 ± 1.7	
	Δ	−1.1 ± 0.8 *^ac^	+2.8 ± 1.1 *^ab^	+7.5 ± 2.0 *^bc^	
Energy (kcal/d)	Basal	2282.6 ± 659.0	2373.4 ± 553.0	2477.7 ± 992.5	0.204
	6-months	2266.5 ± 849.4	2100.3 ± 592.1	2012.2 ± 567.6	
	Δ	−16.0 ± 737.1	−273.0 ± 638.4 *	−465.5 ± 971.9 *	
Vegetables (g/d) per 1000 kcal	Basal	337.0 ± 177.2	320.9 ± 167.1	282.9 ± 176.2	0.547
	6-months	344.9 ± 197.0	375.3 ± 200.8	330.1 ± 121.4	
	Δ	+7.9 ± 132.9	+54.4 ± 143.1 *	+47.2 ± 178.2	
Fruits (g/d) per 1000 kcal	Basal	366.1 ± 175.7	326.1 ± 212.9	246.6 ± 192.2	0.252
	6-months	338.1 ± 197.5	371.4 ± 199.8	359.5 ± 255.0	
	Δ	−28.0 ± 157.4	+45.2 ± 172.1	+112.9 ± 238.8 *	
Legumes (g/d) per 1000 kcal	Basal	23.3 ± 9.2	21.8 ± 13.1	21.0 ± 11.9	0.139
	6-months	26.9 ± 25.3	29.4 ± 17.6	37.3 ± 31.2	
	Δ	+3.6 ± 26.1	+7.6 ± 17.4 *	+16.2 ± 30.6 *	
Cereal (g/d) per 1000 kcal	Basal	140.5 ± 66.1	141.2 ± 72.2	158.4 ± 117.6	0.548
	6-months	134.2 ± 97.0	131.9 ± 63.8	119.9 ± 68.1	
	Δ	−6.3 ± 51.8	−9.3 ± 96.5	−38.4 ± 130.8 *	
Dairy (mL/d) per 1000 kcal	Basal	340.6 ± 243.8	364.5 ± 210.7	301.1 ± 190.2	0.050
	6-months	297.6 ± 148.6	362.5 ± 233.8	397.7 ± 222.5	
	Δ	−43.0 ± 212.9	−2.1 ± 233.6	+96.6 ± 200.9 *	
Meats and meat products (g/d) per 1000 kcal	Basal	151.5 ± 87.2	182.6 ± 75.9	191.9 ± 71.3	0.120
	6-months	139.1 ± 69.8	149.6 ± 61.5	130.7 ± 77.6	
	Δ	−12.3 ± 26.7	−33.0 ± 63.6 *	−61.2 ± 88.7 *	
Olive oil (g/d) per 1000 kcal	Basal	27.5 ± 9.2	32.3 ± 20.2	33.4 ± 20.2	0.358
	6-months	35.9 ± 14.7	29.4 ± 18.5	30.1 ± 17.9	
	Δ	+8.4 ± 16.7	−2.9 ± 22.9	−3.3 ± 23.9	
Fish (g/d) per 1000 kcal	Basal	79.2 ± 52.0	96.9 ± 61.0	96.2 ± 61.0	0.661
	6-months	96.9 ± 87.9	124.8 ± 67.5	123.1 ± 68.5	
	Δ	+17.8 ± 43.6	+27.9 ± 59.1 *	+26.9 ± 68.7 *	
Nuts (g/d) per 1000 kcal	Basal	16.4 ± 16.8	15.6 ± 16.8	8.3 ± 12.0	0.005
	6-months	17.0 ± 21.6	19.5 ± 22.7	29.9 ± 32.2	
	Δ	+0.6 ± 11.9	+3.9 ± 22.0 ^b^	+21.6 ± 30.8 *^b^	
Sweets and pastries (g/d) per 1000 kcal	Basal	31.2 ± 74.3	22.0 ± 23.8	44.3 ± 72.9	0.515
	6-months	22.7 ± 47.8	15.4 ± 22.8	13.1 ± 26.2	
	Δ	−8.5 ± 86.4	−6.5 ± 19.3 *	−31.2 ± 79.8 *	
Soft Drinks (mL/d) per 1000 kcal	Basal	21.8 ± 54.6	25.3 ± 98.9	41.6 ± 95.5	0.002
	6-months	15.4 ± 32.2	2.7 ± 6.9	3.1 ± 5.7	
	Δ	−6.4 ± 37.7 ^ac^	−22.6 ± 99.3 ^a^	−38.4 ± 96.0 *^c^	

Abbreviations: ∆: delta. Data are expressed as mean ± standard deviation (SD). * *p* < 0.05. ^‡^ Differences between tertiles groups at 6 months after adjustment for baseline values by ANCOVA, Post hoc test by Bonferroni; ^a^: differences between No changes in MedDiet adherence and Moderate changes in MedDiet adherence; ^b^: differences between Moderate changes in MedDiet adherence and High changes in MedDiet adherence; ^c^: differences between No changes in MedDiet adherence and High changes in MedDiet adherence.

**Table 4:** Association between the reversion and amelioration of intrahepatic fat contents and the categories of the changes of adherence to MedDiet after 6-month follow-up.

Outcomes		No Changes in Adherence to MedDiet (n = 15)	Moderate Changes in Adherence to MedDiet (n = 56)	High Changes in Adherence to MedDiet (n = 57)
Reversion of IFC by MRI	Crude OR	1.00 (ref)	1.159 (0.332–4.046)	3.822 (1.100–13.281) *
	Adjusted OR	1.00 (ref)	1.514 (0.371–6.175)	4.017 (0.965–16.719)
Amelioration of IFC by MRI	Crude OR	1.00 (ref)	4.235 (1.135–15.799) *	11.250 (2.889–43.809) *
	Adjusted OR	1.00 (ref)	4.925 (1.273–19.046) *	12.995 (3.117–54.187) *

Abbreviations: IFC: Intrahepatic fat contents; OR: Odds Ratio; Adjusted by waist circumference, high blood pressure, and type 2 diabetes mellitus. * *p* < 0.05.

## Data Availability

There are restrictions on the availability of data for this trial, due to the signed consent agreements around data sharing, which only allow access to external researchers for studies following the project’s purposes. Requestors wishing to access the trial data used in this study can make a request to pep.tur@uib.es.
